# Correlation of admitted nursing home residents’ hospital length of stay and vitamin D levels

**DOI:** 10.3402/jchimp.v1i3.6313

**Published:** 2011-10-17

**Authors:** Carla Mc Williams, Kourosh Golestany, Rohit Sharma, Golali Nejati, Anna Cyrus-Murden, Dmitri Kripichnikov

**Affiliations:** Department of Internal Medicine, Lutheran Medical Center, Brooklyn, New York, USA

**Keywords:** vitamin D deficiency, aged, 25-hydroxyvitamin D, nursing homes, dietary supplements

## Abstract

**Objective:**

To determine the relationship between low vitamin D levels and hospital length of stay in nursing home residents who were admitted to acute medical floors in an urban community teaching hospital.

**Methods:**

This prospective cohort study used multiple regression analysis for patients transferred from nursing homes to the hospital. On admission, patients’ serum 25(OH)D levels were obtained by blood draw using partially purified lipid extracts via a competitive protein binding assay. We defined low levels of serum 25(OH)D as <30 ng/ml. Patient medical histories were compiled by retrospective chart review and/or patient interview. Medical histories were analyzed with special emphasis on history of falls, osteoporosis, comorbidities, medication profile, and hospital length of stay.

**Results:**

The mean serum 25(OH)D level for 71 patients (*N* = 71) was 22.69 ng/ml (±SD 10.967); the median, 23 ng/ml. Low serum concentrations of 25(OH)D were recorded in 51 patients (72%) all of whom had a longer mean hospital length of stay (13.72 days ± SD 10.778) than the 20 patients with higher vitamin D levels (7.72 days ± SD 4.070).

**Conclusion:**

Low vitamin D levels in nursing home residents admitted to a community hospital were directly associated with increased hospital length of stay.

The growing prevalence of vitamin D deficiency has been widely documented (1–7), along with its associations of increased risk of type I diabetes, autoimmune disorders, cardiovascular disease, muscle weakness, and functional deficits (1, 2, 8). In addition, studies have indicated that vitamin D deficiency adversely affects recovery in hospitalized patients and potentially increases hospital length of stay (LOS) (3, 9).

Current research has addressed trends in vitamin D deficiency, especially in the institutionalized geriatric populations such as in Drinka et al. (10). Studies have also addressed the increased risk of hip fracture in both institutionalized and healthy independent geriatric populations in vitamin D deficiency such as in Lips et al. (11). However, only a few studies (3, 9) have investigated the correlation between LOS and low levels of serum 25(OH)D and of these studies, none specifically addressed the acute care hospital setting. Moore and Kiebzak (3) addressed deficiency in both acute care and non-hospitalized patients, showing a relationship between increased muscle weakness, deficits, and increased hospital LOS. Kiebzak et al. (9) studied the relationship of vitamin D deficiency and increased rehabilitation LOS.

The goal of this study was to determine whether low levels of serum 25(OH)D were associated with longer hospital LOS in nursing home residents admitted to acute medical floors in an urban community teaching hospital.

## Methods

This prospective cohort study included 71 patients transferred from nursing homes to the hospital. Internal Review Board approval was obtained. On admission, patients’ serum 25(OH)D levels were obtained during a routine phlebotomy using partially purified lipid extracts via a competitive protein binding assay. There was no time limit on when charted serum 25(OH)D levels were last checked nor were adequate oral, tube feeding, or supplemental vitamin D data collected from the nursing home transfer note. Our institution's laboratory defines normal vitamin D levels as 30–72 ng/ml (deficient <19 ng/ml, insufficient = 20–29 ng/ml). Therefore, we defined low levels of serum 25(OH)D as a concentration < 30 ng/ml. Patient medical histories were compiled by retrospective chart review and patient interviews when possible. The medical histories were analyzed with special emphasis on the following variables and comorbidities: history of falls, osteoporosis, fractures, coronary artery disease (CAD), congestive heart failure (CHF), chronic obstructive pulmonary disease (COPD), chronic kidney disease (CKD), cerebrovascular accident (CVA/stroke), hypoparathyroidism, hypertension (HTN), dementia, decubitus ulcers, diabetes mellitus (DM), Parkinson's disease, psychiatric disorders, peripheral vascular disease (PVD), seizures, arrhythmias, hypothyroidism, malignancy, intensive care unit (ICU) admission and transfer, expiration, medication profile, and hospital LOS.

### Statistical analysis

The chi-square and *t*-test were used to assess the relation between low levels of serum 25(OH)D and LOS and demographic and comorbidity variables. Linear regression was done to examine the association between vitamin D level and length of stay adjusting for other covariates related to length of stay. Analyses were conducted by members of the study team using statistical analysis software package SPSS version 18.0.

## Results

Of the 71 patients (mean age 82.2±10.3 years), 49 were women and 22 were men. The mean serum 25(OH)D level was 22.7 ng/ml (±11.0) and median serum 25(OH)D level was 23 ng/ml. Descriptive characteristics of the sample are provided in Table 1. Fifty-one (72%) had low serum concentrations of 25(OH)D (<30 ng/ml). These 51 patients had a longer mean hospital LOS (13.7 days±10.8, ±SD) than patients with normal levels of 25(OH)D (7.7 days±4.1) (*p*=0.002). Nineteen of 22 men (86%) had low levels of serum 25(OH)D levels compared to 32 of 49 (65%) women (*p =* 0.09). Patients in the Intensive Care Unit (ICU) had a high prevalence of low vitamin D levels: 13 (87%) of the 15 were admitted directly to the ICU, and 29 (69%) of the 42 transferred to ICU after admission to a medical floor. More patients with decubitus ulcers (*p =* 0.05) and stroke (*p =* 0.03) had low serum 25(OH)D levels. History of falls, osteoporosis, and the other comorbidities were not related to vitamin D levels (Table 2).

**Table 1:** Descriptive characteristics of the sample

Characteristic	*N*=71
Age (year)	
Mean	81.5
SD	10.9
Range	51–98
Gender	
Male	22 (31.0%)
Female	49 (69.0%)
Hospital length of stay (days) (*n*=65)	
Mean	12.1
SD	9.8
Range	2–62
Calcium (mg/dL)	
Mean	8.8
SD	0.8
Range	7.0–11.0
Phosphorus (mg/dL) (*n*=70)	
Mean	3.4
SD	0.7
Range	2.0–4.9
Magnesium (mg/dL) (*n*=70)	
Mean	2.1
SD	0.3
Range	1.5–2.9
Alkaline phosphatase (IU/L)	
Mean	107.4
SD	106.0
Range	8–835
Total 25 OH-D (ng/ml)	
Mean	22.7
SD	11.0
Range	5–55
Albumin (g/dL)	
Mean	3.3
SD	0.7
Range	1.7–4.9
Urea nitrogen (BUN) (mg/dL)	
Mean	26.9
SD	17.8
Range	5–91
Creatinine (mg/dL)	
Mean	1.1
SD	0.8
Range	0.3–4.1
Coronary artery disease	
Yes	27 (38.0%)
No	44 (62.0%)
Congestive heart failure	
Yes	19 (26.8%)
No	52 (73.2%)
Chronic obstructive pulmonary disease (COPD)	
Yes	15 (21.1%)
No	56 (78.9%)
Falls (history of falls?)	
Yes	8 (11.3%)
No	63 (88.7%)
Cerebrovascular accident (CVA)	
Yes	17 (23.9%)
No	54 (76.1%)
Chronic kidney disease (CKD)	
Yes	7 (9.9%)
No	64 (90.1%)
Osteoporosis	
Yes	14 (19.7%)
No	57 (80.3%)

**Table 2:** Relationship of comorbidities to low vitamin D levels

	Vitamin D levels <30 ng/ml	
		
Comorbidity	*n*/total (%)	*p*
Coronary artery disease		
with	22/27 (82)	0.184
without	29/44 (66)	
Congestive heart failure		
with	16/19 (84)	0.236
without	35/52 (67)	
Chronic obstructive pulmonary disease		
with	10/15 (67)	0.748
without	41/56 (73)	
Falls		
with	6/8 (75)	1.0
without	45/63 (71)	
History of stroke		
with	16/17 (94)	0.028
without	35/54 (65)	
Chronic kidney disease		
with	4/7 (57)	0.394
without	47/64 (73)	
Osteoporosis		
with	10/14 (71)	1.0
without	41/57 (72)	
Fracture		
with	7/12 (58)	0.299
without	44/59 (75)	
Hypoparathyroidism		
with	0/0 (0)	
without	51/71 (72)	*
Hypertension		
with	37/52 (71)	1.0
without	14/19 (74)	
Decubitus ulcer		
with	10/10 (100)	0.053
without	41/61 (67)	
Dementia		
with	27/36 (75)	0.605
without	24/35 (67)	
Diabetes mellitus		
with	15/20 (75)	0.778
without	36/51 (71)	
Parkinson's disease		
with	2/4 (50)	0.314
without	49/67 (73)	
Psychiatric disorder		
with	10/20 (50)	0.018
without	41/51 (80)	
Peripheral vascular disease		
with	2/2 (100)	1.0
without	49/69 (71)	
History of seizures		
with	4/6 (67)	1.0
without	47/65 (72)	
Arrhythmias		
with	10/14 (72)	1.0
without	41/57 (72)	
Hypothyroidism		
with	8/11 (73)	1.0
without	43/60 (72)	
Dyslipidemia medications		
with	9/10 (90)	0.263
without	42/61 (69)	
Malignancy		
with	4/6 (67)	1.0
without	47/65 (72)	

* No *p*-value was calculated because no patient had hypoparathyroidism.

In addition to Vitamin D level, calcium (*r*=–0.301, *p =* 0.011) and albumin (*r*=0–.349, *p =* 0.004) levels were correlated with length of stay. After adjusting for calcium (*p*=0.399) and albumin (*p =* 0.104), vitamin D level was still associated with hospital LOS (*p*=0.005) (Table 3).

**Table 3:** Linear regression model predicative of length of stay

Factors	Beta	*p*
Vitamin D (constant)	—	0.005
Calcium	−0.134	0.399
Albumin	−0.260	0.104

## Discussion

Our study's results suggested that low levels of serum 25(OH)D were associated with increased hospital LOS. This remained valid even after logistic regression with variables such as age and sex, comorbidities, and serum albumin and calcium concentrations. Furthermore, our analysis failed to identify a significant correlation of low vitamin D levels to any particular comorbidity in respect to LOS. Similar results were observed by Kiebzak et al. (9) in their 2007 study: they reported an inverse relationship between serum 25(OH)D levels and rehabilitation unit LOS (9). The higher the patients’ serum 25(OH)D concentrations the shorter their LOS in the hospital rehabilitation unit (9).

Prior studies have suggested that patients with low levels of serum 25(OH)D may have an increased risk of falls, osteoporosis, and fracture, with a subsequent decline in functional status, and more complications from health issues such as obesity, diabetes, and cardiovascular disease (7, 8, 12, 13). Our study, however, found no evidence to support that increased hospital LOS was associated with those particular comorbidities. Comparison of our study population with other studies’ patient populations is difficult to make due to differences in patient care settings and definitions of low vitamin D levels and their various cutoff values (3, 8–10, 14, 15). Our patient population had a high prevalence (72%) of low serum 25(OH)D concentrations, especially in men (86%) compared to women (65%). This may indicate that nursing home residents with low vitamin D levels were prone to develop acute conditions leading to hospital placement. On the other hand, low vitamin D levels may be a predictor, rather than a cause, of increased hospital LOS.

Limitations of the current study included the relatively small sample size and a lack of data regarding patient mobility and dietary intake or supplemental vitamin D in the nursing home. Also, there is known seasonal variability in vitamin D levels. Our data were collected primarily in the fall and winter months, which could have contributed to lower levels as compared to spring or summer. Note that reference ranges for vitamin D levels vary from one laboratory to the next. The cutoff range in our study was <30 ng/ml; however, this number cannot be used in every institution for the aforementioned reasons. Cutoff levels for low vitamin D may need to be addressed on an institutional basis.

## Conclusion

Our results showed that low serum 25(OH) D concentrations in nursing home residents were associated with an increased hospital LOS. Further research is needed to determine whether vitamin D supplementation in this patient population will decrease their number of hospital admissions and/or their LOS during those admissions.

## References

[1] Holick MF (2005). The vitamin D epidemic and its health consequences. J Nutr.

[2] Lee JH, O'Keefe JH, Bell D, Hensrud DD, Holick MF (2008). Vitamin D deficiency: An important, common, and easily treatable cardiovascular risk factor?. J Am Coll Cardiol.

[3] Moore NL, Kiebzak GM (2007). Suboptimal vitamin D status is a highly prevalent but treatable condition in both hospitalized patients and the general population. J Am Acad Nurse Pract.

[4] Ginde AA, Liu MC, Camargo CA (2009). Demographic differences and trends of vitamin D insufficiency in the US population, 1988–2004. Arch Intern Med.

[5] Tangpricha V, Khazai NB (2009). Vitamin D deficiency and related disorders. MedScape.

[6] Chapuy MC, Preziosi P, Maamer M, Arnaud S, Galan P, Hercberg S (1997). Prevalence of vitamin D insufficiency in an adult normal population. Osteoporos Int.

[7] Scragg R, Sowers M, Bell C (2004). Serum 25-hydroxyvitamin D, diabetes, and ethnicity in the Third National Health and Nutrition Examination Survey. Diabetes Care.

[8] Holick MF (2004). Sunlight and vitamin D for bone health and prevention of autoimmune diseases, cancers, and cardiovascular disease. Am J Clin Nutr.

[9] Kiebzak GM, Moore NL, Margolis S, Hollis B, Kevorkian CG (2007). Vitamin D status of patients admitted to a hospital rehabilitation unit: Relationship to function and progress. Am J Phys Med Rehabil.

[10] Drinka PJ, Krause PF, Nest LJ, Goodman BM (2007). Determinants of vitamin D levels in nursing home residents. J Am Med Dir Assoc.

[11] Lips P, van Ginkel FC, Jongen MJ, Rubertus F, van der Vijgh WJ, Netelenbos JC (1987). Determinants of vitamin D status in patients with hip fracture and in elderly control subjects. Am J Clin Nutr.

[12] Bischoff-Ferrari HA, Dawson-Hughes B, Willett WC, Staehelin HB, Bazemore MG, Zee RY (2004). Effect of vitamin D on falls – A meta-analysis. JAMA.

[13] Harris SS, Dawson-Hughes B (2002). Plasma vitamin D and 25OHD responses of young and old men to supplementation with vitamin D3. J Am Coll Nutr.

[14] Steingrimsdottir L, Gunnarsson O, Indridason OS, Franzson L, Sigurdsson G (2005). Relationship between serum parathyroid hormone levels, vitamin D sufficiency, and calcium intake. JAMA.

[15] Holick MF (2007). Vitamin D deficiency. N Engl J Med.

